# Excessive Production of Transforming Growth Factor β1 Causes Mural Cell Depletion From Cerebral Small Vessels

**DOI:** 10.3389/fnagi.2020.00151

**Published:** 2020-06-03

**Authors:** Taisuke Kato, Yumi Sekine, Hiroaki Nozaki, Masahiro Uemura, Shoichiro Ando, Sachiko Hirokawa, Osamu Onodera

**Affiliations:** ^1^Department of System Pathology for Neurological Disorders, Brain Science Branch, Brain Research Institute, Niigata University, Niigata, Japan; ^2^Department of Neurology, Clinical Neuroscience Branch, Brain Research Institute, Niigata University, Niigata, Japan

**Keywords:** TGFβ, mural cells, smooth muscle cells, pericytes, cerebral small vessel

## Abstract

It is increasingly becoming apparent that cerebrovascular dysfunction contributes to the pathogenic processes involved in vascular dementia, Alzheimer’s disease, and other neurodegenerative disorders. Under these pathologic conditions, the degeneration of cerebral blood vessels is frequently accompanied by a loss of mural cells from the vascular walls. Vascular mural cells play pivotal roles in cerebrovascular functions, such as regulation of cerebral blood flow and maintenance of the blood-brain barrier (BBB). Therefore, cerebrovascular mural cell impairment is involved in the pathophysiology of vascular-related encephalopathies, and protecting these cells is essential for maintaining brain health. However, our understanding of the molecular mechanism underlying mural cell abnormalities is incomplete. Several reports have indicated that dysregulated transforming growth factor β (TGFβ) signaling is involved in the development of cerebral arteriopathies. These studies have specifically suggested the involvement of TGFβ overproduction. Although cerebrovascular toxicity *via* vascular fibrosis by extracellular matrix accumulation or amyloid deposition is known to occur with enhanced TGFβ production, whether increased TGFβ results in the degeneration of vascular mural cells *in vivo* remains unknown. Here, we demonstrated that chronic TGFβ1 overproduction causes a dropout of mural cells and reduces their coverage on cerebral vessels in both smooth muscle cells and pericytes. Mural cell degeneration was also accompanied by vascular luminal dilation. TGFβ1 overproduction in astrocytes significantly increased TGFβ1 content in the cerebrospinal fluid (CSF) and increased TGFβ signaling-regulated gene expression in both pial arteries and brain capillaries. These results indicate that TGFβ is an important effector that mediates mural cell abnormalities under pathological conditions related to cerebral arteriopathies.

## Introduction

The neurovascular unit is composed of endothelial cells, vascular mural cells, astrocytes, and neurons and plays a central role in rigorous brain functions. Vascular mural cells have been described as a heterogeneous cell population but are mainly divided into two types of cells, vascular smooth muscle cells (SMCs) and pericytes, which are distinguished by their cellular localization, structure, and gene expression profiling (Holm et al., 2018). Vascular SMCs surround brain pial arteries and arterioles, and pericytes envelope cerebral capillaries. These cells are involved in maintaining precise regulation of cerebral blood flow, blood-brain-barrier (BBB) integrity, and homeostasis of the central nervous system (Armulik et al., 2010; Hall et al., 2014; Hill et al., 2015). Therefore, maintaining the soundness of vascular mural cells is necessary to meet the high energy demand of the brain and BBB function. Impairment or deficiency of vascular mural cells has been reported in some neurodegenerative diseases, including Alzheimer’s disease and amyotrophic lateral sclerosis, and in non-amyloid cerebral small vessel diseases (Ervin et al., 2004; Oide et al., 2008; Winkler et al., 2013). Vascular mural cell loss or dysfunction leads to BBB dysfunction, neuroinflammation, and disrupted coordination between cerebral blood flow and local neuronal activity, ultimately resulting in neuronal loss and dementia. However, the molecular mechanism underlying these impairments of vascular mural cells is unknown.

Transforming growth factor β (TGFβ) signaling promotes cell differentiation, maturation, proliferation, migration, and attachment of endothelial cells and mural cells (Holm et al., 2018). Canonical and normally controlled TGFβ signaling exerts beneficial functions in the vascular milieu. TGFβ signaling promotes the barrier function of the BBB through the upregulation of tight junction proteins (Ronaldson et al., 2009) and induces the differentiation of mural cells. On the other hand, disrupted TGFβ signaling is a common denominator in Alzheimer’s disease and non-amyloid cerebral small vessel disease (Hara et al., 2009). In Alzheimer’s disease, TGFβ protein and its mRNA levels have been reported to be upregulated (Chao et al., 1994; Wyss-Coray et al., 1997). Also, TGFβ protein and mRNA levels are positively correlated with the degree of angiopathy. Moreover, hypertension, which is the strongest risk factor for non-amyloid cerebral small vessel disease, upregulates TGFβ expression. Cerebrovascular accumulation of TGFβ is also observed in hereditary cerebral small vessel disease (Wyss-Coray et al., 1997; Hara et al., 2009; Müller et al., 2017). Although increased TGFβ levels and vascular mural cell abnormalities are important, common characteristics in several cerebral angiopathies, the direct relationships have not been fully investigated.

In this study, we investigated the alterations of vascular mural cells in an environment in which the cells are exposed to excess and long-term TGFβ signaling. To achieve this goal, we used transgenic mice expressing the bioactive form of TGFβ1 (TGFβ1 Tg mice) and assessed the effect on vascular mural cells.

## Materials and Methods

### Animals

In this study, we used transgenic mice overexpressing bioactive porcine TGFβ1 under the control of a glial fibrillary acidic protein (GFAP) promoter (GFAP-TGFβ1 mice; line T64; Wyss-Coray et al., 1995). The animal study was approved by the Animal Use and Care Committee of Niigata University and followed the guidelines of the National Institutes of Health (USA). We maintained and used the Tg mice in the C57BL/6 genetic background as heterozygotes.

### Tissue Preparation

Mice were deeply anesthetized with isoflurane, transcardially perfused with Hank’s balanced salt solution (HBSS), and fixed with 4% paraformaldehyde. For paraffin sections, brains were processed for paraffin embedding. Coronal slices (4 μm) were sectioned from each paraffin-embedded brain block. For vibratome sections, fixed brains were embedded in 3% agarose. Coronal sections (50 μm) were cut on a vibratome.

### Immunohistochemistry

Paraffinized brain sections were rehydrated and then boiled in a microwave oven in 0.01 M sodium citrate buffer (pH 6.0) for antigen retrieval. Brain slices were blocked in 5% fetal bovine serum in PBS + 0.1% Triton X-100 for 1 h at room temperature. Samples were incubated at 4°C with biotinylated anti-α-smooth muscle actin (αSMA) antibody (1:100, LS-C87562, LifeSpan BioScience Inc.), DyLight 594-labeled Lycopersicon esculentum (tomato) lectin (DL-1177, 1:100, Vector Lab.) for visualization of the endothelial cell layer, anti-porcine TGFβ1 antibody (CPT-001, 1:500, Cell Sciences), anti-GFAP antibody (MAB-360, 1:50, Merck Millipore) and anti-Nestin antibody (sc-23927, 1:100, Santa Cruz Biotech.) overnight. Excess antibody was removed by rinsing in PBS. Samples were then incubated at room temperature for 1 h with the secondary fluorescently labeled antibody. Excess antibody was removed by rinsing in PBS. Slides were mounted in Vectashield mounting medium with DAPI (Vector Labs, Burlingame, CA, USA) and imaged with an all-in-one microscope (Keyence; BioRevo BZ-9000). The vessel wall structure was visualized by detecting tissue autofluorescence along with the fluorescently labeled lectin signal.

Vibratome sections were blocked with 5% normal swine serum/1% BSA in PBS containing 0.5% Triton X-100 overnight at 4°C and incubated with rat anti-CD13 antibody (1:50, R3-63, AbD Serotec) with DyLight 594-labeled tomato lectin or rat anti-platelet endothelial cell adhesion molecule-1 (PECAM1) antibody (1:20, DIA-310, Optistain) for 48 h at 4°C. Then, the samples were incubated at 4°C for 24 h with the secondary fluorescently labeled antibody. Three-dimensional fluorescence microscopy images were captured by confocal laser microscopy (LSM710, Carl Zeiss).

### Image Analysis

The occupancy of SMCs in the vascular wall was determined as the ratio of the αSMA-positive area to the vascular wall area. We analyzed micrographic images of cross-sections of the pial artery (anterior cerebral artery) at equal intervals, avoiding arterial branching points. Four to five images were analyzed per mouse using Imaris software (ver. 6.2.0, Bitplane). The size of each SMC was measured using Imaris software. The luminal area of the pial arteries was analyzed using ImageJ software.

To measure pericyte coverage and capillary diameter, three to four three-dimensional fluorescence microscopy images obtained from the motor cortex or the hippocampus were analyzed per mouse. At the time of blood vessel imaging, the region including the parenchymal arterioles was excluded, and only capillaries (~5 μm diameter) were imaged (Ma et al., 2018). Imaris software was used for three-dimensional volume rendering of pericytes and quantification of capillary diameter.

### Quantification of TGFβ1 in Cerebrospinal Fluid (CSF)

Mouse CSF was sampled from the cisterna magna using a glass capillary tube. CSF TGFβ1 was quantified with a Mouse/Rat/Porcine/Canine TGFβ1 Quantikine ELISA Kit (R&D System) according to the manufacturer’s guidelines.

### Pial Artery Collection

Blood was removed by transcardial perfusion with HBSS. The segments of the middle and anterior cerebral arteries with medium-sized branches (referred to as the pial artery) were isolated from mouse brains under a dissecting microscope, immediately frozen on dry ice, and stored at −80°C.

### Brain Capillary Purification

Brain capillaries were purified as previously described (Olson and Soriano, 2011). Cerebral cortexes with the leptomeninges and pial arteries removed were triturated and incubated in 5 mg/ml collagenase type 1 (GIBCO) for 30 min at 37°C. Capillaries were filtered through a 40-μm nylon mesh. The capillaries on the mesh were collected by washing with cold PBS containing 0.1% BSA and 2 mM EDTA and purified from the tissue slurries by affinity purification with anti-PECAM-1 antibody (550274, BD Biosciences) binding magnetic Dynabeads (Thermo Fisher Scientific) for 30 min at 4°C followed by RNA extraction.

### RNA Isolation and cDNA Synthesis

RNA was isolated from the above-collected tissues using a Direct-zol RNA Kit (ZYMO Research). The quantitation and quality of RNA were determined using a Nanodrop 2000c spectrophotometer. Then, RNA from each sample was reverse transcribed to synthesize cDNA using SuperScript IV VILO MasterMix (ThermoFisher).

### Quantitative RT-PCR

For quantitative RT-PCR analysis, reverse-transcribed cDNA was subjected to RT-PCR using the SYBR Green master mix and a Thermal Cycler Dice^®^ Real Time System (Takara).

### Cerebrovascular SMC Culture

Cerebrovascular SMCs isolated from human brains (ScienCell) were maintained in SMC medium (ScienCell) containing 5% FBS at 37°C with 5% CO_2_-95% room air.

### Proliferation Assay

The cell proliferation rate was measured by 5-ethynyl-2′-deoxyuridine (EdU), a thymidine analog, incorporation assay. Following starvation in 1% FBS for 24 h, EdU incorporation into cerebrovascular SMCs was assessed with or without recombinant human TGFβ1 (5 or 50 ng/ml) for 24 h. Recombinant TGFβ1 was dissolved in 4 mM HCl with 2% BSA. After EdU incorporation, cells were stained with a Click-iT EdU Imaging Kit (Thermo Fisher Scientific) according to the manufacturer’s instructions.

### Cell Death Assay

For the cell death assay, cerebrovascular SMCs were treated with recombinant human TGFβ1 (5 or 50 ng/ml) for 3 days. Dying cells were detected using an Apoptosis/Necrosis Assay Kit (Abcam) according to the manufacturer’s instructions. As a positive control for this assay, cerebrovascular SMCs were also treated with camptothecin (6 μM).

### Statistical Analysis

Statistical computation was performed using IBM SPSS 22. Data were first subjected to the Shapiro–Wilk test (for fit to the Gaussian distribution) and Levene’s test (for equal variance). Either a one-way analysis of variance or a two-tailed unpaired *t*-test was adopted for data with a Gaussian distribution and equal variance. Subsequently, a Bonferroni test was applied to the data as a *post hoc* test. Alternatively, the Mann–Whitney *U*-test was applied to data with unequal variance. *P* < 0.05 was regarded as statistically significant.

### Study Approval

All animal experiments described were approved by the Animal Use and Care Committee of Niigata University and followed the guidelines of the National Institutes of Health (USA).

## Results

**Figure 1:**
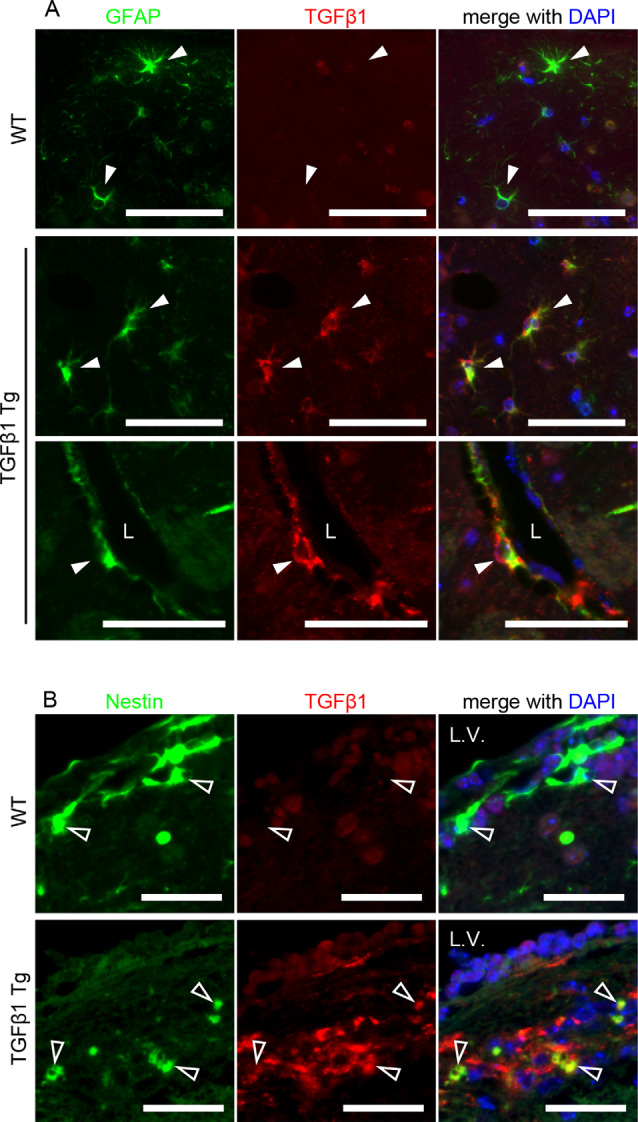
Cellular distribution of porcine transforming growth factor β (TGFβ1) transgene expression. **(A)** Brain slices of 8-week-old TGFβ1 Tg mice were double-labeled with glial fibrillary acidic protein (GFAP) and porcine TGFβ1 antibodies. TGFβ1 immunoreactivity was specifically detected in astrocytes of TGFβ1 Tg mice but not wild-type (WT) mice. The overexpression of porcine TGFβ1 was also observed in perivascular astrocytes (lower images). Filled white arrowheads depict GFAP-positive astrocytes. L, vascular lumen. Scale bar = 50 μm. **(B)** Porcine TGFβ1 expression in Nestin-positive cells around the lateral ventricle was detected in the brains of 8-week-old TGFβ1 Tg mice. L.V., lateral ventricle. Empty arrowheads depict Nestin-positive cells. No detectable signal of TGFβ1 expression was found in the age-matched WT brain slices. Scale bar = 25 μm.

**Figure 2:**
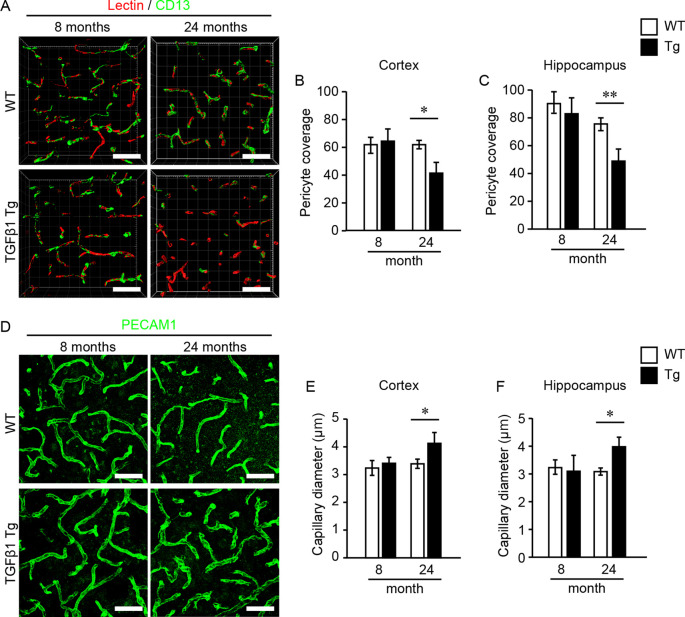
Pericyte coverage analysis and quantification of brain capillary diameter. Pericyte coverage on brain capillaries and capillary diameter were quantified in 8- and 24-month-old mouse brain samples. **(A)** Representative 3D volume-rendered images of endothelial cells (red) and pericytes (green) in the cerebral cortex. The analysis was conducted using images obtained from the cerebral cortex **(B)** and the hippocampus **(C)**. Bar graphs show the results of quantifications of pericyte coverage in each brain region. Three images were analyzed in each brain region per mouse. **(D)** Representative images of endothelial cells depicted by PECAM1 immunostaining in the cerebral cortex. The bar graphs show the mean capillary diameter in the cerebral cortex **(E)** and the hippocampus **(F)** at 8 and 24 months of age. Two images were analyzed in each brain region per mouse. Data represent the mean ± SE, *P < 0.05 and **P < 0.01 according to a two-tailed unpaired *t*-test (*n* = 4–5 animals per group). Scale bar = 30 μm.

First, we investigated pericytes on brain capillaries that were directly enveloped by the endfeet of astrocytes overexpressing TGFβ1 in Tg mice at 8 and 24 months of age. Indeed, TGFβ1 overexpression was confirmed in astrocytes in the brains of TGFβ1 Tg mice (Figure 1A). Also, overexpression of TGFβ1 was observed in Nestin-positive neural stem cells, which are a subpopulation of astrocytes that express GFAP and Nestin, around the lateral ventricle (Figure 1B; Gonzalez-Perez and Quiñones-Hinojosa, 2012). The pericyte coverage rate on the capillary walls was significantly lower in both the cerebral cortex and the hippocampus in TGFβ1 Tg mice than in wild-type (WT) mice at 24 months of age. The decrease in pericyte coverage in TGFβ1 Tg mice was not found at 8 months of age (Figures 2A–C). A decrease in pericyte coverage has been associated with the expansion of the capillary vessel diameter (Armulik et al., 2010). We examined capillary diameter by morphometric analysis of immunostained capillary endothelial cells and found that the capillary diameter in TGFβ1 Tg mice was significantly larger than that in WT mice only at 24 months of age but not at 8 months of age (Figures 2D–F).

**Figure 3:**
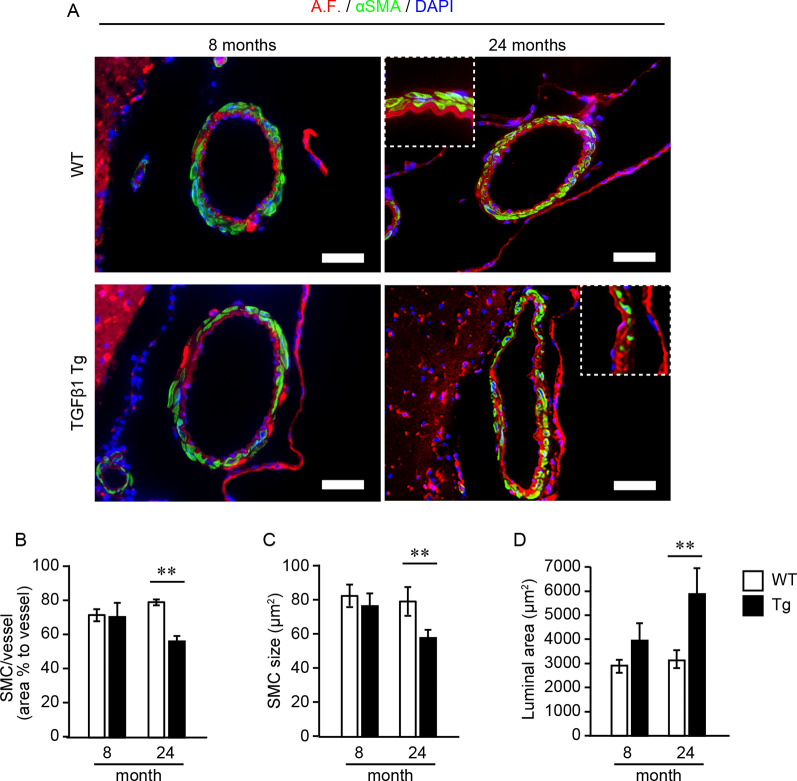
Analysis of smooth muscle cell (SMC) occupancy in pial arteries and morphometric analysis. **(A)** Representative images of pial arteries depicted by anti-α-smooth muscle actin (αSMA) immunostaining (green) and tissue autofluorescence (A.F.). Each bar graph shows the SMC area occupancy to the area of the vascular wall **(B)**, the mean size of each SMC **(C)**, and the luminal area **(D)** in 8- and 24-month-old mice. Data represent the mean ± SE, **P < 0.01 according to a two-tailed unpaired *t*-test (*n* = 4–5 animals per group).

Next, we investigated the effect of TGFβ1 overexpression on vascular SMCs in brain pial arteries by immunohistochemistry at 8 and 24 months of age. The entire circumference of the pial arteries in WT mice was covered by stratified SMCs, even at 24 months of age. In contrast, SMC loss from the vascular walls was frequently observed in TGFβ1 Tg mice at 24 months of age, resulting in a decreasing coverage rate of SMCs on the vascular walls (Figures 3A,B). At 24 months of age, the mean size of the remaining SMCs was also significantly smaller in TGFβ1 Tg mice than in age-matched WT mice (Figures 3A,C). Morphologically, the luminal area of the pial arteries was significantly larger in TGFβ1 Tg mice (Figures 3A,D). These abnormalities were not found in the pial arteries of 8-month-old TGFβ1 Tg mice (Figures 3A–D).

Vascular SMCs mainly lie on the pial arteries and the perforating arteries.

**Figure 4:**
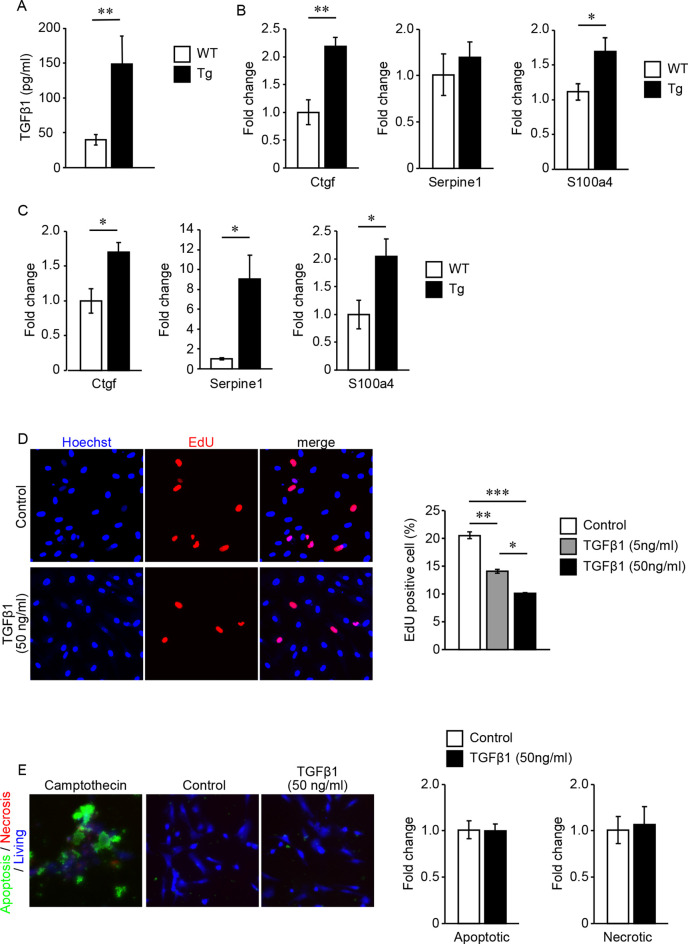
**(A–C)** Quantification of TGFβ1 content in the cerebrospinal fluid (CSF) and gene expression levels regulated by TGFβ1 signaling. **(A)** TGFβ1 content in the CSF of 7-month-old TGFβ1 Tg mice and WT mice. **P < 0.01 according to the Mann-Whitney *U*-test (*n* = 3 animals per group). **(B,C)** Gene expression levels regulated by TGFβ1 signaling in pial arteries **(B)** and brain capillaries **(C)** of 7-month-old TGFβ1 Tg mice and WT mice. *P < 0.05 and **P < 0.01 according to a two-tailed unpaired *t*-test (*n* = 5 animals per group). **(D)** Cell proliferation rate under treatment with vehicle or recombinant TGFβ1 was detected by an EdU incorporation assay. Representative images of vehicle- (4 mM HCl with 2% BSA) or 50 ng/ml recombinant TGFβ1-treated cells are shown. Recombinant TGFβ1 repressed the proliferation rate of cerebrovascular SMCs in a dose-dependent manner (*n* = 5 independent experiments). **(E)** Cerebrovascular SMCs treated with recombinant TGFβ1 or camptothecin (6 μM). Inducible apoptotic, necrotic, and living cells were detected by a phosphatidylserine sensor (green), 7-AAD (red), and cytocalcein violet 450 (blue), respectively. Apoptotic and necrotic cell signal areas were quantified and normalized by the area of the living cell signal. Data are represented as a relative value to non-treated control cells. *P < 0.05, **P < 0.01 and ***P < 0.001 according to Bonferroni’s multiple comparison *post hoc* test (*n* = 5 independent experiments). Data represent the mean ± SE.

However, these types of brain vessels lack direct contact with astrocytes because the pial arteries or perforating arteries and astrocytes (astrocytic glia limitans) are separated by the subarachnoid space or the perivascular space, which contains CSF. Quantification of active TGFβ1 content in the CSF revealed that active TGFβ1 content was upregulated in TGFβ1 Tg mice compared with WT mice (Figure 4A). We also quantified the expression levels of mRNA regulated by TGFβ signaling using samples of dissected pial arteries and purified brain parenchymal small vessels. In both types of cerebral vessels, the expression levels of genes regulated by TGFβ signaling were significantly upregulated in TGFβ1 Tg mice compared with WT mice (Figures 4B,C).

The effects of TGFβ signaling on vascular mural cells have been reported in several studies. However, the response to signaling depends on the state of gene expression in cells receiving this signal (Grainger et al., 1994; Suwanabol et al., 2012). Because cerebral blood vessels have specialized gene expression profiling, we examined the direct response of cerebrovascular SMCs to TGFβ signaling (Jambusaria et al., 2020). EdU incorporation assay revealed that TGFβ1 exerts a dose-dependent inhibitory effect on cerebrovascular SMC proliferation (Figure 4D). On the other hand, even high concentrations of TGF did not induce cell death of vascular SMCs (Figure 4E).

## Discussion

We demonstrated that the constitutive overproduction of TGFβ1 from astrocytes results in the degeneration of vascular mural cells. While various cell types express TGFβ in the central nervous system, the main sources of increased TGFβ under pathological conditions are astrocytes and microglia (Finch et al., 1993; Buckwalter and Wyss-Coray, 2004; Yan et al., 2014). TGF production increases with aging as well as vascular injury (Yan et al., 2014). Astrocytes highly interact with cerebral blood vessels by enwrapping through their endfeet. Thus, the increased TGFβ production from astrocytes should more directly and strongly affect blood vessels than TGFβ production from other cell types. The adverse effect is extended not only to parenchymal pericytes surrounded by astrocyte endfeet but also to SMCs that were not in direct contact with astrocytes. Our analysis of mouse CSF revealed that TGFβ1 content was upregulated in TGFβ1 Tg mice. The entire subarachnoid space is sealed by the astrocytic glia limitans superficialis. We assume that TGFβ1 secreted from superficial glia limitans affects SMCs in pial arteries. Indeed, in concurrence with gene expression levels in the capillaries, gene expression levels regulated by TGFβ signaling were higher in the pial arteries of TGFβ1 Tg mice than in those of WT mice. The lowering of mural cell coverage was accompanied by dilation of brain vessels, which is compatible with reports of pericyte-deficient model mice (Armulik et al., 2010).

We speculate that the suppression of mural cell proliferation by TGFβ signaling is involved in the mechanism of the decrease in mural cell coverage rates of cerebral blood vessels in TGFβ1 Tg mice. It has been reported that the proliferation of vascular SMCs, which are derived from peripheral blood vessels, is suppressed by TGFβ treatment (Grainger et al., 1994; Martin-Garrido et al., 2013). In this study, we observed a similar inhibitory effect on the proliferation of brain-derived SMCs without cell death. Maintenance of medial SMC number involves the proliferation of resident vascular SMCs. Vascular SMC death induces cell proliferation of adjacent SMCs, and the portion that lost SMCs is repaired (Yu et al., 2011). The decreased mural cell coverage of cerebral blood vessels and the degenerative alteration may be due to impairment of cell proliferation-related tissue maintenance mechanisms. Our results that mural cell alterations manifest with age are compatible with this interpretation. Also, in an *in vitro* study of some progenitor or stem cells, TGFβ signaling has an inhibitory effect on the proliferation of these cells (Larsson et al., 2003). In this study, we found overexpression of TGFβ1 in Nestin-positive neural progenitor cells in TGFβ1 Tg mice. Indeed, reduced proliferation and fewer neural progenitor cells have been reported in TGFβ1 Tg mice (Buckwalter et al., 2006). As another source of vascular SMCs, local adventitial vascular SMC progenitors are known in peripheral blood vessels (Majesky et al., 2011). Whether these cells are present in cerebral blood vessels remains unknown, but the effect on the self-proliferation of progenitor cells may also be related to the results obtained here.

Wyss-Coray et al. (1997) reported that astrocytic TGFβ1 overproduction in human β-amyloid precursor protein (hAPP) mice exacerbated cerebral amyloid angiopathy. Aβ exerts cell toxicity against vascular mural cells. In the monogenic TGFβ1 Tg mice used in this study, thioflavin S-positive amyloid is also deposited to the basement membrane of cerebral vessels (Wyss-Coray et al., 2000). Although the substances composing the amyloid deposition in monogenic TGFβ1 Tg mice are currently unknown, this amyloid deposition may be involved in mural cell degeneration. In addition to amyloid deposition, increases in basement membrane proteins, perlecan, and fibronectin have been observed in the cerebral vessels of TGFβ1 Tg mice (Wyss-Coray et al., 2000). The accumulation of extracellular matrix proteins precedes amyloid deposition. The brain drainage system flows from the brain parenchyma along the basement membrane to the lymph nodes. Therefore, altered basement membrane compositions may impede the elimination of toxic waste products and lead to harmful deposition of these products in cerebral vessels.

Notably, these accumulated extracellular matrix proteins identified in TGFβ1 Tg mice have been observed in both hereditary and sporadic non-amyloid cerebral small vessel disease (Nag and Kilty, 1997; Zellner et al., 2018). Conventionally, blood pressure overload, and high blood glucose levels have been noted to increase the expression of extracellular matrix proteins and cause vascular accumulation under these pathological conditions. In recent years, it has become clear that extracellular matrix abnormalities in hereditary non-amyloid cerebral small vessel disease also occur through sequestration of regulator protein or proteolytic dysregulation (Monet-Leprêtre et al., 2013; Zellner et al., 2018). Extracellular matrix abnormalities have attracted attention as a major factor in the molecular pathogenesis of cerebral small vessel disease. In considering treatments, it is imperative to understand how TGFβ signaling participates in extracellular matrix abnormalities in various cerebral arteriopathies (Humphrey et al., 2014).

We demonstrated that TGFβ1 overproduction causes a dropout of mural cells from the vascular wall and decreases the coverage of small vessels. However, a long amount of time is needed for this phenotype to become apparent. The major limitation of this study is that it remains unclear whether the two phenomena are directly linked *in vivo*. Canonically, TGFβ signaling promotes the development of blood vessels and induces mural cells around blood vessels at the developmental stage. However, TGFβ signaling exerts context-dependent effects. In particular, the effect depends on the status of downstream signaling effectors, including their receptors (Armulik et al., 2005). TGFβ signaling also has pleiotropic effects on immune systems. For example, TGFβ signaling engages in crosstalk with interleukin-6 (IL-6) in orchestrating inflammatory responses. In vascular systems, IL-6 has the potential to increase pericyte coverage. Enhanced TGFβ signaling may inhibit the effect of IL-6 on promoting pericyte coverage by attenuating IL-6 signaling (Ricard et al., 2014; Wiegertjes et al., 2019). In addition to the immune system, TGFβ engages in crosstalk with various signal cascades that function in the vasculature, such as platelet-derived growth factor and vascular endothelial growth factor. We assume that not a single but multiple mechanisms are involved in the process of mural cell abnormalities in TGFβ1 Tg mice. Further research is required to clarify these issues.

In summary, our analysis using TGFβ1 Tg mice provides a link between the increased production of TGFβ and the degeneration of mural cells, both of which are observed in several cerebral arteriopathies. Mural cell degeneration has serious effects on brain functions that require high spatiotemporal regulation of blood flow. While further exploration is needed, our results indicate that TGFβ signaling may be a therapeutic target for protecting mural cell degeneration.

## Data Availability Statement

The datasets generated for this study are available on request to the corresponding author.

## Ethics Statement

The animal study was reviewed and approved by the Animal Use and Care Committee of Niigata University.

## Author Contributions

The image analysis was performed by YS and TK. The biochemical analysis was performed by YS, SA, and TK. Animal care was provided by SH. MU, HN, and OO designed the project. TK and OO prepared the manuscript.

## Conflict of Interest

The authors declare that the research was conducted in the absence of any commercial or financial relationships that could be construed as a potential conflict of interest.
